# Achievement of treatment goals for secondary prevention of myocardial infarction or stroke in 29,325 patients with type 2 diabetes: a German/Austrian DPV-multicenter analysis

**DOI:** 10.1186/s12933-016-0391-8

**Published:** 2016-05-03

**Authors:** Barbara Bohn, Christof Schöfl, Vincent Zimmer, Michael Hummel, Nikolai Heise, Erhard Siegel, Wolfram Karges, Michaela Riedl, Reinhard W. Holl, On behalf of the DPV-initiative

**Affiliations:** Institute of Epidemiology and Medical Biometry, ZIBMT, University of Ulm, Albert-Einstein-Allee 41, 89081 Ulm, Germany; Division of Endocrinology and Diabetes, Department of Medicine I, University Hospital Erlangen, Friedrich-Alexander-University, Erlangen-Nuremberg, Germany; Department for Internal Medicine, Protestant Hospital Zweibrücken, Zweibrücken, Germany; Department of Medicine II, Saarland University Medical Center, Homburg, Germany; Specialized Diabetes Practice Rosenheim & Institute of Diabetes Research, Helmholtz Center Munich, Munich, Germany; Alb Fils Kliniken, Helfenstein Clinic, Geislingen, Germany; Department of Internal Medicine, St. Josefs Hospital, Heidelberg, Germany; Division of Endocrinology and Diabetes, RWTH Aachen University, Aachen, Germany; Division of Endocrinology and Metabolism, Department of Internal Medicine III, Medical University of Vienna, Vienna, Austria; German Center for Diabetes Research (DZD), Munich-Neuherberg, Germany

**Keywords:** Type 2 diabetes, Stroke, Myocardial infarction, Secondary prevention, Guideline adherence

## Abstract

**Background:**

To analyze whether medical care is in accordance with guidelines for secondary prevention of myocardial infarction (MI), or stroke in patients with type 2 diabetes from Germany and Austria.

**Methods:**

29,325 patients (≥20 years of age) with type 2 diabetes and MI, or stroke, documented between 2006 and 2015 were selected from the Diabetes-Patienten-Verlaufsdokumentation database. We analyzed medication, clinical characteristics, and lifestyle factors according to national secondary prevention guidelines in patients with MI, or stroke, separately.

**Results:**

HbA_1C_ <7.5 % was achieved in 64.9 % (MI), and in 61.1 % (stroke) of patients. LDL <100 mg/dl was documented in 56.2 % (MI), and in 42.2 % (stroke). Non-smoking was reported in 92.0 % (MI), and in 93.1 % (stroke), physical activity in 9.6 % (MI), and 5.5 % (stroke). Target values of blood pressure (<130/80 mmHg in MI, 120/70–140/90 in stroke) were reached in 67.0 % (MI), and in 89.9 % (stroke). Prescription prevalence of inhibitors of platelet aggregation (IPA) was 50.7 % (MI), and 31.7 % (stroke). 57.0 % (MI), and 40.1 % (stroke) used statins, 65.1 % (MI), and 65.8 % (stroke) used any type of antihypertensives, and ACE inhibitors were prescribed in 49.7 % (MI), and 41.3 % (stroke). A body mass index (BMI) <27 kg/m^2^ and the use of beta blockers were only recommended in subjects with MI. Of the patients with MI, 32.0 % had a BMI <27 kg/m^2^, and 59.5 % used beta blockers.

**Conclusions:**

Achievement of treatment goals in secondary prevention of MI, or stroke in subjects with type 2 diabetes needs improvement. Target goals were met more frequently in patients with MI compared to subjects with stroke. Especially the use of IPA was very low in patients with stroke. There remains great potential to reduce the risk of repeated macrovascular events and premature death, as well as to increase patients’ quality of life.

## Background

The prevalence of type 2 diabetes is increasing worldwide [1–3]. Moreover, studies indicate an earlier onset of type 2 diabetes and a longer life-expectancy with diabetes [2, 4]. A prolonged exposure to an adverse diabetic milieu and a high prevalence of cardiovascular risk factors (as obesity, hypertension, or dyslipidemia) [5], contribute to a high risk for macrovascular complications (MVCs) as myocardial infarction (MI), or stroke. In patients with diabetes, cardiovascular disease (CVD) is two to eight folds higher compared to the general population and is the leading cause of death [6–8]. Additionally, disease progression in patients with type 2 diabetes seems to be more severe with a worse long-term prognosis compared to subjects without diabetes [5, 9]. MVCs have an adverse effect on patients’ quality of life and are also a huge public health problem due to high economic costs [10–12].

German and international guidelines (from e.g. the American Heart Association) for secondary prevention of MI and stroke aim to reduce cardiovascular morbidity and mortality and to improve patient’s quality of life [8, 13–15]. These guidelines address pharmacological and lifestyle interventions, and provide target values amongst others for serum glucose, blood pressure, or serum lipids [8, 13–15]. However, data of previous studies indicate a suboptimal implementation of guidelines in medical care [16–21]. Although there are studies analyzing primary prevention of CVD risk factors in patients with diabetes [21–25], or adherence to secondary prevention guidelines in the general population [26–29] no studies exist investigating the achievement of current guideline targets for secondary prevention of MVCs in subjects with type 2 diabetes.

Our objective was to examine whether medical care in patients with type 2 diabetes who already experienced a MI or stroke meets current guideline recommendations for secondary prevention. We also analyzed sociodemographic and clinical differences between patients with and without MVCs.

## Methods

### Data source and subjects

Data were provided by the German/Austrian DPV (Diabetes-Patienten-Verlaufsdokumentation) registry. The DPV software is used for standardized, prospective documentation of diabetes care and outcome and is currently used by 428 centers from Germany (n = 398) and Austria (n = 30). Twice a year, data are anonymized and transmitted from the participating health care facilities to Ulm, Germany, and aggregated into a cumulative database for clinical research and quality assurance [16]. Implausible and inconsistent data are reported back to the centers for verification or correction. The DPV initiative is approved by the Ethics Committee of the University of Ulm, Germany and data collection by the local review boards.

**Fig. 1:**
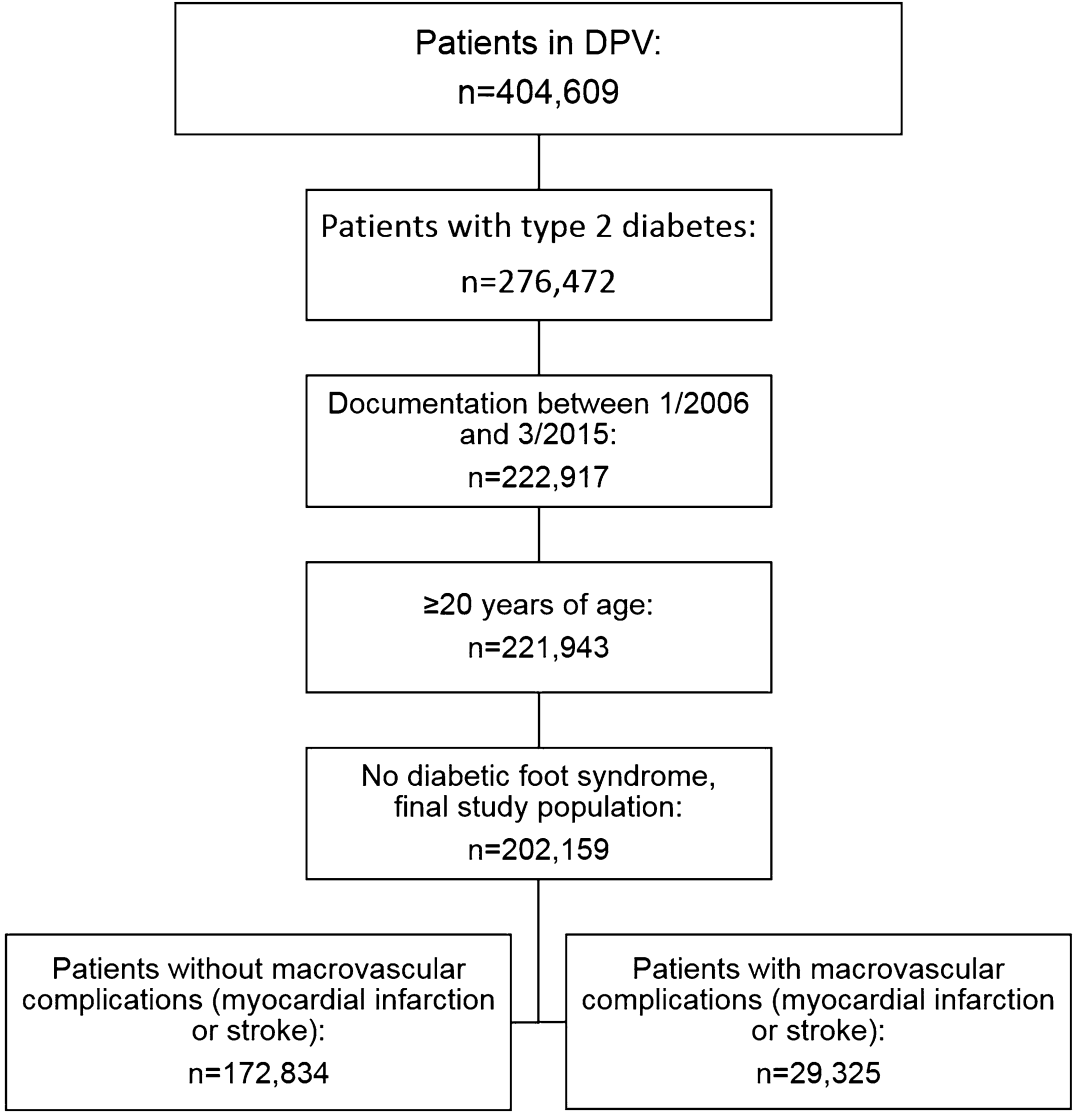
Selection of study population

As of March 2015, 404,609 patients were registered in DPV. Adult patients (≥20 years of age) with type 2 diabetes documented during the year 2006 or thereafter were included; leaving 221,943 subjects from 178 participating centers (Fig. 1). If a macrovascular complication (stroke, or MI) was documented in the DPV software by the physician, the patient was assigned to the respective patient group (“MI only”, “stroke only”, “MI and stroke”). The diabetic foot syndrome (DFS) is a result of macrovascular and polyneuropathic complications. Additionally, guidelines for DFS contain recommendations primarily for wound management [30]. Hence, patients with DFS were excluded from the present analysis (n = 19,784; Fig. 1).

Due to heterogeneity on medical possibilities, and available drugs, data before the year 2006 were excluded. The recommendations considered did not differ between the year 2006 and 2015. For each patient, the last year of treatment was analyzed.

### Outcomes

Sociodemographic characteristics (as e.g. sex, age, and age at diagnosis), clinical data (HbA_1C_, BMI, serum lipids, systolic and diastolic blood pressure), and lifestyle factors (smoking, physical activity) were compared between patients with at least one MVC (MI, stroke, or both) or patients without MVCs.

We considered variables according to national guidelines. In patients with MI, we analyzed HbA_1C_ (<7.5 %), blood pressure (<130/80 mmHg), LDL-cholesterol (<100 mg/dl), body mass index (BMI) (<27 kg/m^2^), medical treatment (inhibitors of platelet aggregation (IPA), statins, beta blockers, ACE inhibitors, and any type of antihypertensives), smoking (yes/no), and physical activity (yes/no) [13].

In patients with stroke, we analyzed HbA_1C_ (<7.5 %), blood pressure (systolic 120–<140 mmHg/diastolic 70–<90 mmHg), LDL-cholesterol (<100 mg/dl), medical treatment (IPA, statins, ACE inhibitors, and any type of antihypertensives), smoking (yes/no), and physical activity (yes/no) [14, 15].

HbA_1C_ was mathematically standardized to the reference range of 20–42 mmol/mol (diabetes control and complication trial: 4.05–6.05 %) by applying the multiple-of-the-mean transformation method [31]. Systolic and diastolic blood pressure as well as serum lipids were measured in local laboratories compliant with national guidelines [32]. Information on smoking behavior and physical activity in supervised sports groups were based on patient self-reports to their diabetes-care teams.

### Statistical analysis

Sociodemographic characteristics and clinical data were presented as median (Q1;Q3), or as percentage. To compare groups, Chi square (χ^2^) test was used for dichotomous variables, and Kruskal–Wallis test for continuous variables. The false discovery rate (FDR) was applied to correct p-values for multiple comparisons [33]. To analyze potential gender-differences in drug use, clinical characteristics, and recommended lifestyle, logistic regression models were applied in “MI only” and “stroke only” groups. Due to differences in age distribution among men and women, data were adjusted for age-groups (20–<65, 65–<75, >75 years of age).

Due to the large number of subjects studied, a two-sided p value <0.01 was considered significant. All statistical analyses were implemented with SAS 9.4 (Statistical Analysis Software, SAS Institute, Cary, NC, USA).

## Results

### Differences between patients with or without MVCs

We included a total of 202,159 type 2 diabetes subjects (male: 51.4 %) with a median age of 70.6 (Q1;Q3: 60.6;77.9) years and a median diabetes duration of 8.1 (2.7;14.1) years. Of the patients included, 29,325 had at least one MVC. In subjects with MVCs, there was a male preponderance (57.6 vs. 50.3 %; p < 0.0001), their median age was higher (73.6 vs. 69.9 years; p < 0.0001) and the diabetes duration was longer (9.8 vs. 7.8 years; p < 0.0001) compared to patients without MVCs (Table 1). Total cholesterol as well as LDL-cholesterol were higher in patients without MVCs (both p < 0.0001). Differences between patient groups are described in detail in Table 1.

**Table 1:** Comparison of sociodemographic and clinical characteristics between type 2 diabetes patients with and without MVC

	Patients with type 2 diabetes without macrovascular complication (MI, or stroke) (n = 172,834)		Patients with type 2 diabetes and at least one macrovascular complication (MI, or stroke) (n = 29,325)		p*
	n		n		
Men, %	172,834	50.3	29,325	57.6	<0.0001
Age (years)	172,834	69.9 (59.6;77.5)	29,325	73.6 (66.5;79.8)	<0.0001
Age at diagnosis (years)	172,834	59.1 (49.2;68.6)	29,325	62.2 (52.7;70.5)	<0.0001
Diabetes duration (years)	172,834	7.8 (2.6;13.8)	29,325	9.8 (4.2;16.0)	<0.0001
BMI (kg/m^2^)	152,737	29.9 (26.3;34.5)	26,044	29.0 (25.8;32.9)	<0.0001
HbA_1C_ (%)	156,314	7.1 (6.2;8.4)	27,091	6.9 (6.2;8.2)	<0.0001
Systolic BP (mmHg)	161,233	130.0 (120.0;144.0)	28,368	130.0 (120.0;140.0)	<0.0001
Diastolic BP (mmHg)	161,073	80.0 (70.0;80.0)	28,345	75.0 (70.0;80.0)	<0.0001
Total Chol (mg/dl)	122,629	187.0 (156.0;221.0)	23,645	171.0 (143.0;205.0)	<0.0001
LDL-Chol (mg/dl)	109,060	111.0 (85.0;139.0)	22,328	99.0 (76.0;128.0)	<0.0001
HDL-Chol (mg/dl)	111,496	43.0 (35.0;54.0)	22,389	42.0 (34.0;51.0)	<0.0001
Physical inactivity, %	59,410	90.0	11,293	92.5	<0.0001
Smoking, yes, %	121,303	12.5	23,448	9.4	<0.0001
Cigarettes per day	15,173	14 (10;20)	2213	15 (10;20)	0.0975

Data are medians (Q1;Q3) unless otherwise indicated

* p values adjusted for multiple comparisons by FDR

### Differences within patients with MVCs

We also analyzed differences in sociodemographic and clinical characteristics between type 2 diabetes patients with MI only (n = 15,015), stroke only (n = 11,738), or both MVCs (MI and stroke) (n = 2572) (Table 2). The lowest percentage of women was present in patients with MI only (37.0 %), and the highest in patients with stroke only (49.9 %). Age at diabetes diagnosis was earlier in patients with MI (60.9 years) compared to the other groups (e.g. 63.9 years in patients with stroke). Diabetes duration was longest in patients with both MVCs (11.0 years) (Table 2). Diastolic and systolic blood pressure were higher in patients with stroke (135/80 mmHg) compared to patients with MI (130/74 mmHg) or both MVCs (130/75 mmHg). Total- and LDL-cholesterol were highest in patients with stroke. A comprehensive description of patient characteristics can be found in Table 2.

**Table 2:** Comparison of sociodemographic and clinical characteristics between type 2 diabetes patients with different MVCs

	MI only (n = 15,015)		Stroke only (n = 11,738)		MI and stroke (n = 2572)		p*
	n		n		n		
Men, %	15,015	63.0	11,738	50.1	2572	60.6	<0.0001
Age (years)	15,015	72.4 (65.1;78.8)	11,738	74.8 (67.9;81.0)	2572	74.8 (68.5;80.4)	<0.0001
Age at diagnosis (years)	15,015	60.9 (51.4;69.3)	11,738	63.9 (54.6;72.0)	2572	61.8 (52.0;70.0)	<0.0001
Diabetes duration (years)	15,015	9.6 (3.9;16.2)	11,738	9.6 (4.2;15.2)	2572	11.0 (6.2;19.2)	<0.0001
BMI (kg/m^2^)	13,887	29.2 (26.0;33.1)	9902	28.7 (25.5;32.7)	2255	29.1 (25.7;32.9)	<0.0001
HbA_1C_ (%)	13,795	6.9 (6.2;8.1)	10,889	7.0 (6.2;8.3)	2407	7.1 (6.3;8.3)	<0.0001
Systolic BP (mmHg)	14,576	130.0 (120.0;140.0)	11,296	135.0 (120.0;149.0)	2496	130.0 (120.0;140.0)	<0.0001
Diastolic BP (mmHg)	14,562	74.0 (70.0;80.0)	11,287	80.0 (70.0;80.0)	2496	75.0 (70.0;80.0)	<0.0001
Total Chol (mg/dl)	12,750	166.0 (139.2;197.2)	8808	181.0 (150.0;216.0)	2087	169.0 (140.0;203.0)	<0.0001
LDL-Chol (mg/dl)	12,086	94.0 (73.0;121.0)	8245	107.9 (82.0;138.0)	1997	96.0 (73.0;126.0)	<0.0001
HDL-Chol (mg/dl)	12,176	41.0 (34.0;50.0)	8212	43.0 (35.0/52.0)	2001	41.0 (34.0;50.0)	<0.0001
Physical inactivity, %	5217	90.4	4869	94.5	1207	93.3	<0.0001
Smoking, yes, %	12,049	10.0	9189	8.8	2210	9.2	0.0173
Cigarettes per day	1200	15 (10;20)	809	15 (10;20)	201	15 (10;20)	0.1579

Data are medians (Q1;Q3) unless otherwise indicated

* p values adjusted for multiple comparisons by FDR

### Achievement of target goals and medication in patients with type 2 diabetes and MI

**Fig. 2:**
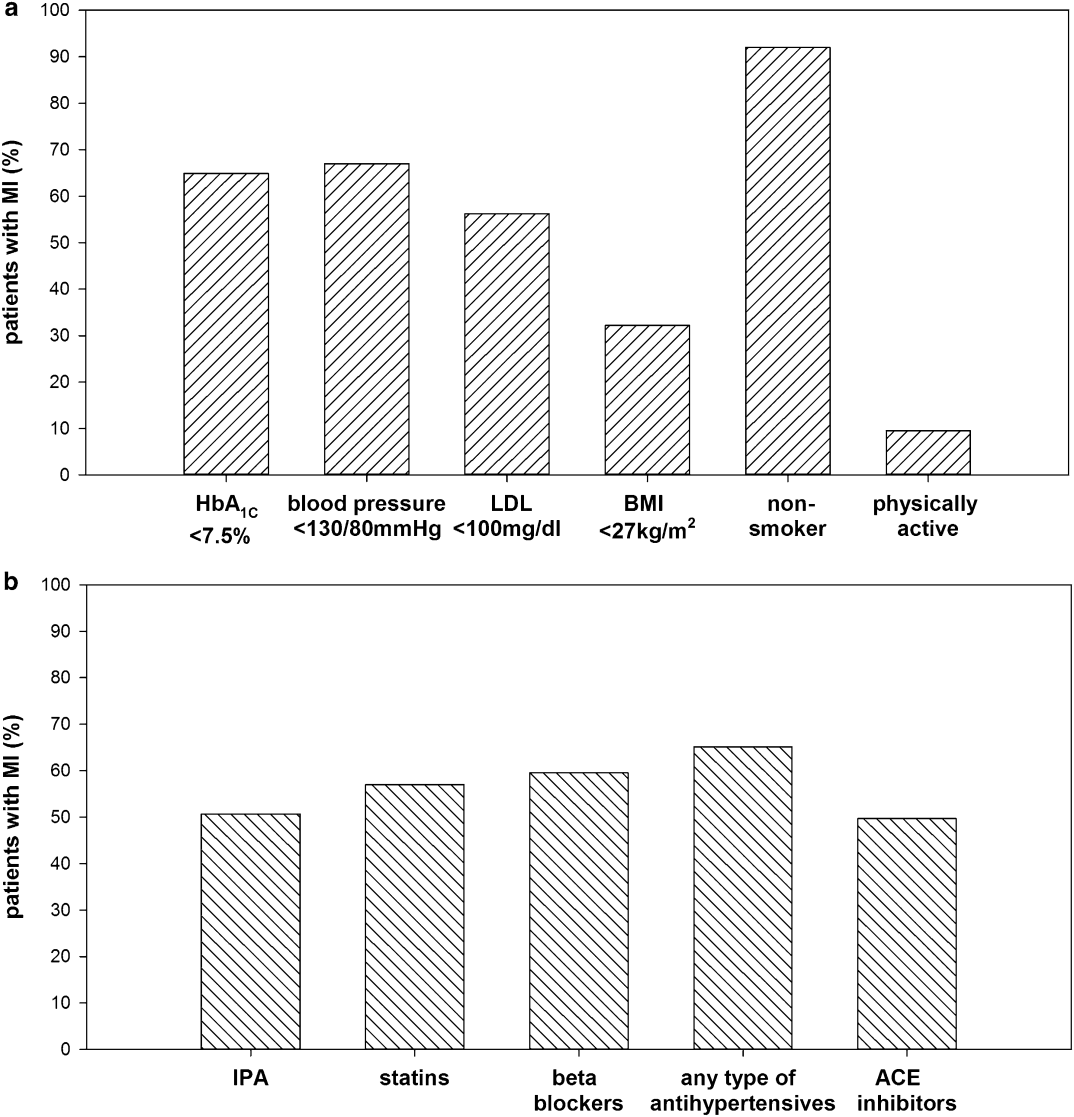
Achievement of treatment goals in patients with type 2 diabetes and myocardial infarction *MI*. **a** Cardiovascular risk control values and lifestyle factors **b** medications

Approximately two-thirds of the patients with type 2 diabetes and MI achieved target values of HbA_1C_ (64.9 %) and blood pressure (67.0 %) (Fig. 2a). More than half of the patients (56.2 %) reached target value of LDL-cholesterol. Guidelines also recommend weight loss in subjects with a BMI over 27 kg/m^2^. In our patients with MI, only one-third (32.2 %) had a BMI of less than 27 kg/m^2^. Physical activity was self-reported by 9.6 % and non-smoking by 92.0 % of the patients (Fig. 2a). Use of different pharmaceutical agent classes as per recommendation was documented in 50 % (use of IPA) to 65 % (use of any type of antihypertensives) of patients (Fig. 2b). Of those patients treated with antihypertensives (n = 9773), 66.6 % reached target values of blood pressure. In subjects treated with statins (n = 8556), 62.8 % achieved LDL <100 mg/dl.

### Achievement of target goals and medication in patients with type 2 diabetes and stroke

**Fig. 3:**
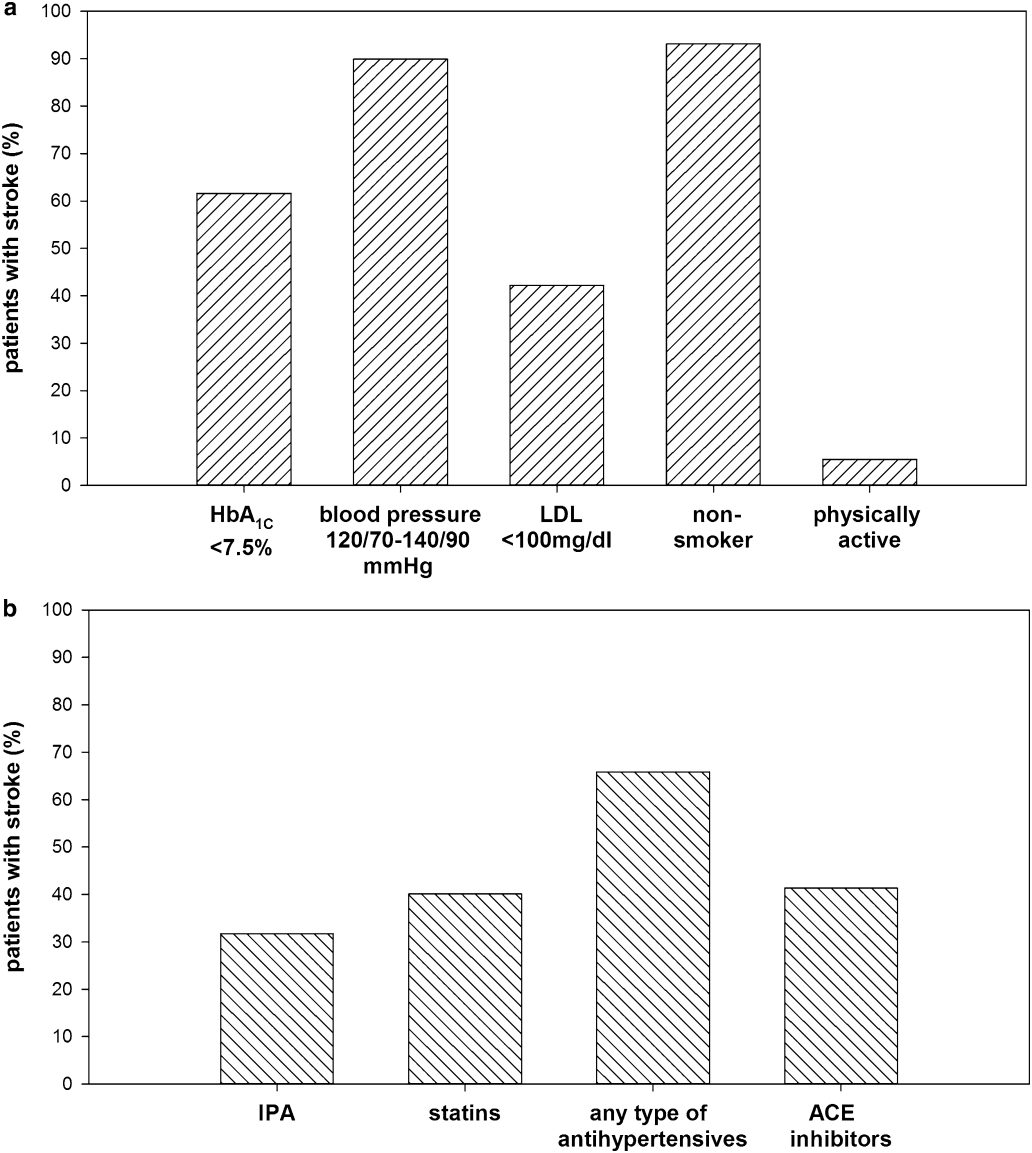
Achievement of treatment goals in patients with type 2 diabetes and stroke. **a** Cardiovascular risk control and lifestyle factors **b** medications

In most patients with stroke (89.9 %), target values of blood pressure were reached. Less than two-thirds had an HbA_1C_ of <7.5 % (Fig. 3a). LDL-cholesterol under 100 mg/dl was only documented in 42.2 %. 5.5 % of the patients reported to be physically active, 93.1 % were documented as non-smokers (Fig. 3a). About two-thirds of the patients were treated with any type of antihypertensive drugs, whereas the use of IPA, statins or ACE inhibitors was only documented in 30 % to 40 % of the subjects with stroke (Fig. 3b). In patients treated with antihypertensives (n = 7726), 89.3 % met a blood pressure between 120/70 and 140/90 mmHg. 49.8 % of statin-treated patients (n = 4710) reached target value of LDL <100 mg/dl.

### Gender differences in the attainment of guideline recommendations

Logistic regression analysis revealed a slightly, but significantly better achievement of recommended treatment goals in men compared to women (Table 3). The biggest difference was observed in LDL-cholesterol. In subjects with MI, 60.1 % of men and 49.2 % of women reached the target value of <100 mg/dl (p < 0.0001). In patients with stroke, 46.1 % of men and 38.3 % of women met the goal. Only the proportion of non-smokers was higher in women compared to men (MI: 94.0 vs. 92.0 %; stroke: 95.3 vs. 92.4 %; both p < 0.0001). Table 3 summarizes all gender-differences.

**Table 3:** Gender differences in the attainment of guideline recommendations, separately for MI and stroke

	MI only			Stroke only		
	Men (n = 9455)	Women (n = 5560)	p*	Men (n = 5883)	Women (n = 5855)	p*
HbA_1C_ <7.5 %, %	65.8	64.1	0.0553	62.2	61.4	0.4082
Blood pressure <130/80 (mmHg), %	67.4	66.5	0.2679	–	–	–
Blood pressure 120/70–140/90 (mmHg), %	–	–	–	90.0	89.5	0.1583
LDL-cholesterol <100 (mg/dl), %	60.1	49.2	<0.0001	46.1	38.3	<0.0001
BMI <27 (kg/m^2^), %	33.5	28.6	<0.0001		–	–
Inhibitors of platelet aggregation, %	51.3	49.4	0.0137	32.7	30.5	0.0118
Statins, %	58.5	54.5	<0.0001	42.0	38.2	<0.0001
ACE inhibitors, %	51.3	46.9	<0.0001	42.2	40.3	0.0414
Beta blockers, %	59.7	59.0	0.4290	–	–	–
Any type of antihypertensives, %	65.9	63.8	0.0115	65.1	66.7	0.0684
Non-smoker, %	92.0	94.0	<0.0001	92.4	95.3	<0.0001
Physically active, %	9.5	8.5	0.2201	5.8	4.7	0.0887

* Data adjusted for age-groups (20–<65, 65–<75, >75 years of age)

## Discussion

Our aim was to analyze the adherence to guidelines for secondary prevention of MI or stroke in patients with type 2 diabetes who already experienced a MI, or stroke. Overall, concordance with recommendations is rather mixed. Achievement of target values was best for glycaemic control and blood pressure. Almost all patients reported to be a non-smoker. Hardly anyone was physically active in supervised sport groups. Even use of medication according to guidelines was quite poor, except for the use of antihypertensives. Treatment goals were met more often in subjects with MI compared to subjects with stroke. Particularly the use of IPA was very low in patients with stroke. We further investigated sociodemographic and clinical characteristics in patients with and without MVCs.

### Differences between patients with or without MVCs

Our analysis revealed lower rates of CVD risk factors in patients with MVCs (Table 1). Especially blood lipids and diastolic blood pressure were lower in patients with MI, or stroke. One explanation for the differences found is that in our analysis, patients with MVCs more frequently receive lipid-lowering drugs (45.1 vs. 23.8 %) or antihypertensives (65.8 vs. 48.7 %) compared to subjects without MVCs. It can be also assumed that patients with a history of at least one MVC are more concerned about their health. Furthermore it is possible that medical care is more intensive in subjects with MVCs and physicians might be more focused on CVD risk factors. This assumption can be supported by results of the German DETECT study, indicating that prescription rates of guideline recommended medication in subjects with CVD correlate with the number of comorbidities [28].

### Meeting target goals in patients with MI or stroke

The best achievement was present for blood pressure. In patients with MI, about two-thirds had a blood pressure below 130/80 mmHg (Fig. 2a). In patients with stroke, over 90 % reached recommended values (Fig. 3a). Differences in achievement may be explained by higher target values for stroke patients (70–90 mmHg for diastolic; 120–140 mmHg for systolic blood pressure). In German type 2 diabetes guidelines, target value for blood pressure is 140/80 mmHg [34]. This objective was achieved in 68.6 % of our study population without MVCs.

Studies investigating secondary prevention of MI or stroke in patients with type 2 diabetes are lacking. However, there are studies analyzing the prevalence of CVD risk factors in patients with diabetes, irrespective of previous MVC events [22, 24, 25]. Compared to results of the ESTHER-study, a German cohort study from Saarland (population-based data on primary care; patients with diabetes, n = 1375), achievement of recommended blood pressure targets in the DPV study population is high. In the ESTHER-study, only 8.7 % reached a blood pressure below 130/80 mmHg [24]. Findings from the German DIAB-CORE project (pooled data from six population-based studies; patients with type 2 diabetes, n = 1287), reported in 36.4 % of the subjects a blood pressure below 140/90 mmHg [25]. In an analysis of the National Health and Nutrition Examination Survey (NHANES) from the USA, in the years 2007–2010 (n = 1376), blood pressure of <130/80 mmHg was achieved in 51.1 % of the patients, and values <140/90 mmHg in 72.0 % [22]. Results of the EUROASPIRE IV (European Action on Secondary and Primary Prevention by Intervention to Reduce Events) study, including patients of 24 European countries, reported in 32 % of the patients with newly diagnosed diabetes and in 26 % of the patients with known diabetes a blood pressure <130/80 mmHg [35].

In guidelines for secondary prevention of MI/stroke, normoglycemia is recommended without a specific cut off value [13–15]. According to general national type 2 diabetes guidelines, HbA_1C_ should be between 6.5 and 7.5 % depending on patients’ needs and preferences [34]. We therefore set a threshold of <7.5 %. An HbA_1c_ <7.5 % was documented in approximately two-thirds of the patients with stroke (61.6 %), or MI (64.9 %). This is comparable to subjects without MVCs (60.1 %). A recently published study from Wales analyzed changes in HbA_1C_ levels in patients with diabetes before and 1 year after stroke [36]. The authors reported a significant improvement in HbA_1C_ levels from 7.7 to 7.3 %. They also compared the achievement of HbA_1C_ levels ≤7.5 % in diabetes patients with and without stroke. One year after the incident stroke, an HbA_1C_ ≤7.5 % was documented in 62.5 % of patients who experienced stroke and in 65.3 % of the controls [36]. Our findings are also in line with results of the NHANES study. These authors reported an HbA_1C_ of <7.0 % in 52.5 % of the patients, and an HbA_1C_ of <8.0 % in 77.9 % [22].

In our study population, achievement of LDL targets was worst. Values below 100 mg/dl were present in only 42.2 % of the patients with stroke (Fig. 3a), and in 56.2 % of the patients with MI (Fig. 2a). LDL <100 mg/dl is also recommended in type 2 diabetes without MVCs [34]. Even in these subjects, only 38.8 % met the goal. Hence, high values of LDL might be an overall problem in type 2 diabetes. However, in a direct comparison with other studies from Germany, clinical characteristics of DPV-patients are better. For example in the ESTHER-study, only in 13.3 % of the patients, LDL <100 mg/dl was reported [24]. In the DIAB-CORE study, only information on the mean LDL was available. The mean LDL-value of 137 mg/dl is much higher compared to our study population (105 mg/dl in patients with MVCs, 115 mg/dl in patients without MVCs) [25]. In the US, 56.2 % achieved LDL <100 mg/dl [22]. The EUROASPIRE IV study indicated in 56 % of the patients with newly diagnosed diabetes and in 66 % with known diabetes LDL values <97 mg/dl [35]. Overall, patients included in the current DPV study seem to have better control of CVD risk compared to subjects of other German cohorts [24, 25, 37]. Patients in DPV are mainly treated in specialized diabetes centers, whereas participants of the ESTHER study or the DIAB-CORE project are predominantly treated by general practitioners [24, 25]. This could be one explanation for better treatment. Additionally, benchmarking with other centers participating in the DPV initiative might further encourage the diabetes team to improve clinical care in their patients [38].

### Lifestyle factors

In the DPV database, the documentation of smoking was missing in 19.8 % (n = 2966) with MI, and in 21.7 % (n = 2549) with stroke. Most patients (>90 %) reported to be a non-smoker. This roughly correspondents to the prevalence in the general population. According to the German census, smoking prevalence ranges from 22.0 % in the 60 to <65 year olds to 5.2 % in subjects over 75 years of age [39]. The low smoking prevalence reported in diabetes subjects should be assessed positively, however, under-reporting cannot be completely excluded.

Physical activity is also recommended in the secondary prevention of MI and stroke. In the present analysis, 5 to 10 % of patients with MI or stroke reported to be physically active (Figs. 2a, 3a). In the ESTHER-study, physical activity was reported in 43.6 % [24]. However, in DPV, only physical activity in guided sport-groups is documented by the diabetes team. This could be one explanation for the lower percentage. Physical activity in guided sport-groups is important directly after the first episode after MI/stroke. Since we analyze the last treatment year of the patients, it is possible, that the MI/stroke already dates back several years. Hence, guided sport-groups are no longer required and it is possible that patients exercise privately. Furthermore, physical immobility due to stroke could be a limiting factor in physical activity. Moreover, in the DPV database, information on physical activity is available in only 34.7 % (MI) and 41.5 % (stroke) of patients.

Recommendations on BMI are present only in guidelines for MI. According to national guidelines [13], body weight should be reduced if the BMI is over 27 kg/m^2^. In our analysis, 32.2 % had a BMI <27 kg/m^2^. In patients without MVCs, the proportion is slightly lower (29.9 %). A study from Sweden demonstrated the importance of successful weight management in newly diagnosed patients with type 2 diabetes [40]. The authors stated that overweight and obese subjects had a substantially increased risk of incident atrial fibrillation compared to normal weight subjects. Even modest weight gain during the first 1.5 years after diabetes diagnosis seemed to be associated with increased atrial fibrillation risk [40].

### Medication

Except for antihypertensive drugs (MI: 65.1 %; stroke: 65.8 %), concordance with recommendations is very low in our study population (Figs. 2b, 3b). Treatment goals are met more frequently in patients with MI compared to subjects with stroke. The use of IPA (31.7 %) is especially low in patients with stroke. As already mentioned, we analyzed the last year of treatment. It is therefore possible that recommendations were observed initially, but treatment was discontinued over time. In a large analysis of a German sickness fund (n = 30.028), initial prescription prevalence after myocardial infarction and treatment after 5 years were evaluated [29]. Initial prescription was higher compared to our analysis. Beta blockers were prescribed in 82 % of the patients, statins in 73 %, and ACE inhibitors in 69 % [29]. However, 5 years after a MI, only few of the subjects were still treated according to recommendations with 36 % of the patients prescribed beta-blockers, 31 % ACE inhibitors, and in 17 % statins [29]. A very large study with over half a million subjects from the UK Biobank highlighted that medication was in many people not according to secondary prevention recommendations [26]. A study from the USA analyzed treatment in 364 subjects who survived coronary heart disease [41]. Best adherence to recommendations was present for lipid lowering drugs (60 %), followed by beta blockers (58 %) and ACE inhibitors (38 %) [41]. Other European countries, such as the Netherlands [27], and Denmark [42] have reported a low proportion of subjects meeting guideline-defined treatment goals. To improve secondary prevention of CVD, the introduction of the “polypill” (fixed-dose combination pill) is discussed [43]. The polypill concept was recently implemented in a number of European countries and in the USA [43].

### Subgroup analysis

In almost all cases, treatment goals were met more often in men compared to women (Table 3). Some studies confirm our findings [28, 44, 45], while others do not [23, 24, 46]. For example, the analysis of Xin Song and colleagues indicated in all categories of anthropometric measures of obesity a higher CVD mortality in men compared to women [46]. A suboptimal achievement of target values was also present in those patients treated with antihypertensives or statins—except of blood pressure in patients with stroke. Only half of the statin-treated subjects with MI (49.8 %) and less than two-thirds with stroke (62.8 %) achieved LDL <100 mg/dl. In patients with MI treated with antihypertensives, only about two-thirds (66.6 %) achieved recommended blood pressure values of <130/80 mmHg. This gives rise to the assumption that medication is underdosed in clinical practice. Underdose in CVD medication was confirmed by other studies [47, 48]. Steinberg et al. stated that less than half of the subjects with acute coronary heart syndrome reached target values of LDL <100 mg/dl and that the recorded statin doses were lower than those with an evidence-based effect [48]. This was also reported by a study from the Euro Heart Survey on Diabetes and the heart [47]. The authors indicated that neither dosage of antihypertensive drugs nor dosages of statins were increased despite blood pressure and serum lipids exceeding recommended targets [47]. Although there are many studies indicating an improvement in survival and quality of life in patients who received medical treatment according to guidelines [21, 49, 50], a significant discrepancy between recommendations and actual care is present. There are many potential reasons for this gap. Aside from a lack of knowledge of current guidelines, physician inexperience, lack of physician time, or patients’ non-adherence, clinical reasons such as comorbidities or adverse effects of medications might also be present [18, 21, 51]. Furthermore, external constraints for example the type of health insurance (private vs. statutory health insurances), access to medical care (including distance to the medical practice, or availability of specialists), or financial aspects may also be implicated [18, 21, 51]. Another reason could be a lack of resources available to implement secondary prevention measures [21].

### Strengths and limitations

The main strength of the current observational study is its large number of patients. Since we solely consider subjects from centers participating in the DPV initiative, a selection bias cannot be completely excluded and the generalizability of our results might be therefore limited. Another shortcoming is that we could only assess the proportion of patients meeting treatment goals and not the reasons for non-adherence. A further limitation is that clinical characteristics and medical treatment were not completely documented in all patients with MI, or stroke. However, up to now, there seems to be no comparable study investigating adherence to current secondary prevention guidelines of MI, or stroke in the high-risk group of type 2 diabetes subjects. Despite these limitations, analyses as the present one are urgently needed to reveal weaknesses in medical care providing an evidence basis for improvement.

## Conclusion

This cross-sectional study indicates that medication and lifestyle changes for secondary prevention in subjects with type 2 diabetes who already experienced MI, or stroke are not in line with guideline recommendations. Overall, patients with stroke less frequently met treatment goals compared to subjects with MI. Especially the use of IPA was low. This analysis also confirms the need to improve secondary prevention of CVD risk factors. Aside from greater efforts by physicians to implement current guidelines, new strategies need to be developed to further increase patient motivation and compliance to hopefully result in sustainable behavior changes, including increased physical activity and healthy food choices.

## Abbreviations

BMI: body mass index
CVD: cardiovascular disease
DPV: diabetes patienten verlaufsdokumentation
DSF: diabetic foot syndrome
EUROASPIRE: European Action on Secondary and Primary Prevention by Intervention to Reduce Events
FDR: false discovery rate
IPA: inhibitors of platelet aggregation
MI: myocardial infarction
MVC: macrovascular complication
NHANES: National Health and Nutrition Examination Survey
